# Development and validation of a multidimensional predictive model for 28-day mortality in ICU patients with bloodstream infections: a cohort study

**DOI:** 10.3389/fcimb.2025.1569748

**Published:** 2025-07-07

**Authors:** Jun Jin, Lei Yu, Qingshan Zhou, Qian Du, Xiangrong Nie, Hai-Yan Yin, Wan-Jie Gu

**Affiliations:** ^1^ Department of Intensive Care Unit, The University of Hong Kong-Shenzhen Hospital, Shenzhen, China; ^2^ Department of Intensive Care Unit, The First Affiliated Hospital of Jinan University, Guangzhou, China

**Keywords:** bloodstream infections, predictive model, nomogram, 28-day all-cause mortality, sepsis, intensive care unit, MIMIC-IV database

## Abstract

**Background:**

Bloodstream infections (BSI) are a leading cause of sepsis and death in intensive care unit (ICU). Traditional severity scores, including the Sequential Organ Failure Assessment (SOFA), Acute Physiology Score III (APSIII), and Simplified Acute Physiology Score II (SAPS II), exhibit limitations in effectively predicting mortality among BSI patients, primarily due to their reliance on a narrow range of clinical variables. This study aimed to develop and validate a comprehensive nomogram model for 28-day all-cause mortality prediction in BSI patients.

**Methods:**

A retrospective cohort study was conducted using data from 3,615 patients with positive blood cultures from the MIMIC-IV database, divided into training (n=2,532) and validation (n=1,083) cohorts. Through a two-step variable selection process combining LASSO regression and Boruta algorithm, we identified 12 predictive variables from 58 initial clinical parameters. The model’s performance was evaluated using AUROC, net reclassification improvement (NRI), integrated discrimination improvement (IDI), and decision curve analysis (DCA).

**Results:**

The nomogram demonstrated superior discrimination (AUROC: 0.760 *vs*. 0.671, P<0.001 for SOFA; 0.760 *vs*. 0.705, P<0.001 for APSIII; 0.760 *vs*. 0.707, P<0.001 for SAPS II) in the training cohort, with consistent performance in the validation cohort (AUROC: 0.742). Key predictors identified in our model included the need for mechanical ventilation, the presence of malignancy, platelet count, and scores on the Glasgow Coma Scale (GCS). The model showed significant improvements in NRI and IDI, with consistent net benefit across a wide range of threshold probabilities in DCA.

**Conclusions:**

This study developed and validated a predictive model for 28-day mortality in BSI patients that demonstrated superior performance compared to traditional severity scores. By integrating clinical, laboratory, and treatment-related variables, the model provides a more comprehensive approach to risk stratification. These findings highlight its potential for improving early identification of high-risk patients and guiding clinical decision-making, though further prospective validation is needed to confirm its generalizability.

## Introduction

Bloodstream infections (BSI) are a major precipitant of sepsis and a significant contributor to mortality in intensive care unit (ICU) worldwide (Wittekamp et al., 2018; Grumaz et al., 2020). Patients with BSI face a heightened risk of adverse outcomes, making early identification and targeted management essential for improving survival rates (Zengin Canalp and Bayraktar, 2021). Traditional severity scores, including the Sequential Organ Failure Assessment (SOFA), Acute Physiology Score III (APSIII), and Simplified Acute Physiology Score II (SAPS II), are commonly employed to evaluate the severity of illness in patients with sepsis. However, these scores have limitations in accurately predicting mortality, particularly in patients with BSI, as they rely on a limited set of clinical variables and may not fully capture the unique pathophysiology of BSI-related sepsis.

Sepsis, characterized by a dysregulated immune response to infection, often leads to life-threatening organ dysfunction (Singer et al., 2016; Cecconi et al., 2018; Meyer and Prescott, 2024), with BSI being a common and severe precipitant. The rising incidence of sepsis, particularly cases involving BSI, underscores the need for more precise risk stratification tools. Current predictive models often fail to account for the distinct clinical and laboratory profiles of BSI-related sepsis, highlighting the importance of a more comprehensive approach. Traditional severity scores may not capture the full spectrum of sepsis pathophysiology, especially in the context of BSI (Tian et al., 2016).

Using data from the Medical Information Mart for Intensive Care (MIMIC) database (Johnson et al., 2023), this study aims to develop and validate a predictive model for 28-day all-cause mortality in patients with positive blood cultures. By incorporating multidimensional patient data, we seek to enhance the accuracy of mortality prediction in this high-risk population. The proposed model has the potential to serve as a valuable clinical tool, enabling early identification of high-risk BSI patients and facilitating targeted interventions to improve outcomes.

## Materials and methods

### Data source

The data utilized in this study were extracted from the Medical Information Mart for Intensive Care IV (MIMIC-IV) version 3.0 database. This openly accessible repository contains comprehensive medical information from the ICU of the Massachusetts Institute of Technology Beth Israel Deaconess Medical Center (Johnson et al., 2023), covering patient stays between 2008 and 2022. Permission to use the database was obtained (Certificate No.: 56161429).

### Study population

**Figure 1:**
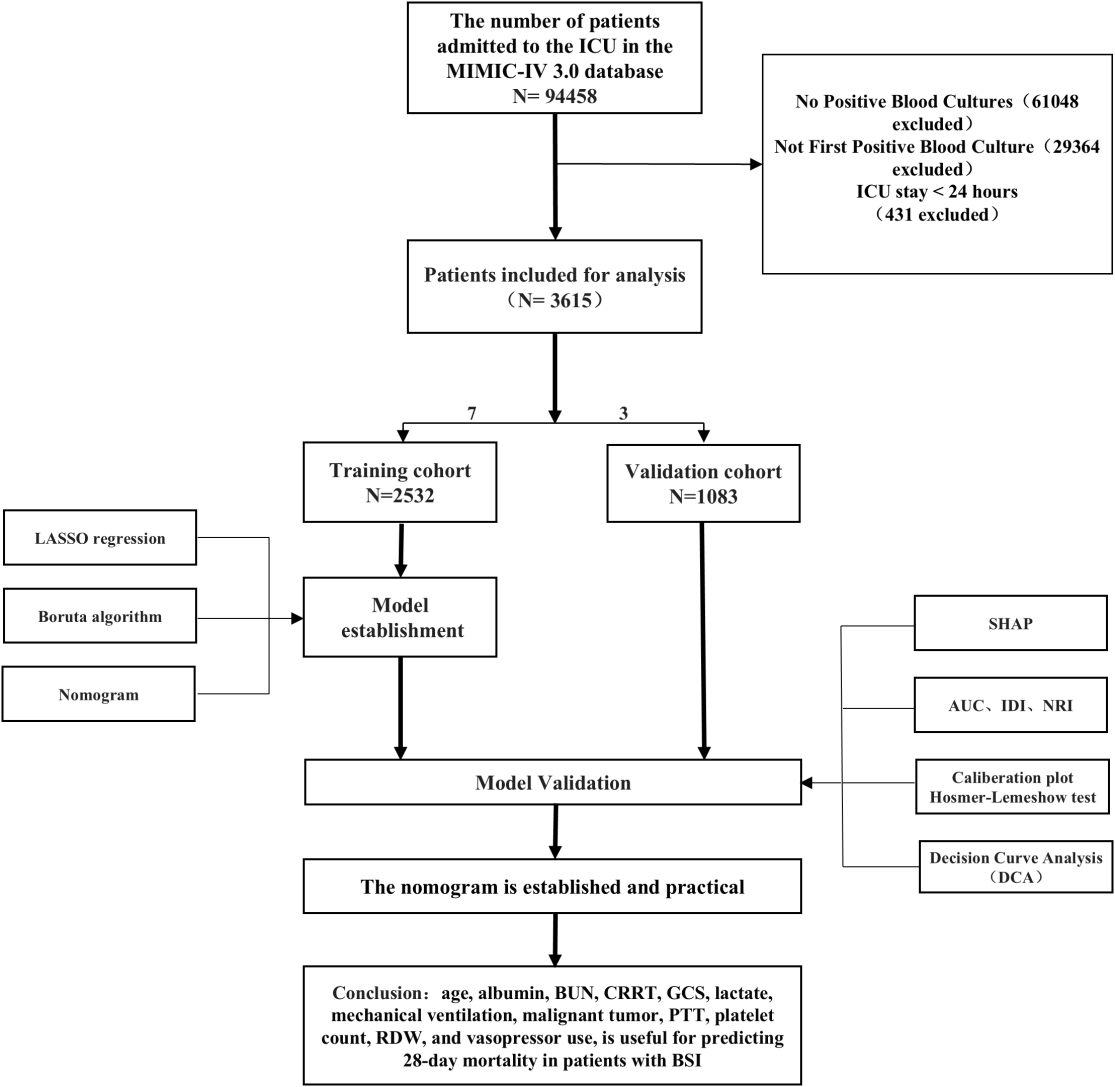
Overall study flowchart.

The study population comprised adult patients (≥18 years) admitted to the ICU for the first time with positive blood cultures, hospital stays exceeding 24 hours, and complete data on key variables. Patients younger than 18 years, those with hospital stays shorter than 24 hours, those with missing data on key variables, and those not admitted to the ICU for the first time were excluded (**Figure 1**).

### Study methods

A total of 58 variables were acquired using SQL (Wu et al., 2021), encompassing baseline data (age, gender, race, BMI, hypertension, diabetes mellitus, malignant tumor, CKD, cirrhosis, heart failure, myocardial infarction, hyperlipidemia, COPD), vital signs (heart rate, systolic blood pressure, diastolic blood pressure, mean arterial pressure, respiratory rate, pulse oximetry, temperature), laboratory tests (GCS, white blood cell count, red blood cell count, platelet count, hemoglobin, RDW, albumin, sodium, potassium, chloride, glucose, pH, partial pressure of carbon dioxide, partial pressure of oxygen, lactate, prothrombin time, PTT, international normalized ratio, total bilirubin, alanine aminotransferase, aspartate aminotransferase, BUN, creatinine), infection and treatment (microorganism, CRRT, MV, vasopressor, midazolam, dexmedetomidine, propofol), outcome measures (length of stay in hospital, length of stay in ICU, in-hospital mortality, ICU mortality), and severity scores (SOFA, APSIII, SAPS II, Charlson Comorbidity Index).

### Statistical methods

#### Data splitting and imputation

For variables with less than 30% missing values, multiple imputations were performed using a regression model. This method was chosen based on the understanding that maintaining a threshold of 30% for missing data helps ensure that imputation methods yield valid and reliable results, thereby minimizing the risk of bias. The imputation process involved iteratively predicting and filling in missing values for each variable, resulting in five complete datasets. One of these datasets was then randomly selected for the final analysis (Zhang, 2016; El Badisy et al., 2024). The research subjects were then randomly assigned into a training set (70%) and a validation set (30%).

#### Variable selection

The variable selection process was conducted on the training set to ensure the robustness and accuracy of the predictive model. Initially, LASSO regression was employed to identify significant predictive factors. The optimal value of the regularization parameter λ was determined through 10-fold cross-validation using the 1-standard error (1-SE) criterion, which helps prevent overfitting by selecting a simpler model that retains predictive power. This approach enhances the model’s interpretability and stability, ensuring that only the most meaningful variables are included. Variables with coefficients significantly different from zero (considering the applied penalty) were shortlisted (Hu et al., 2021). Subsequently, the Boruta algorithm was applied to further refine the variable selection process. This algorithm compares the importance of each variable with that of a randomly permuted copy of itself, ensuring that only those variables demonstrating significantly higher importance than their randomized counterparts are selected. In this process, only the “confirmed” variables from Boruta were retained, providing a robust measure of significance (Kursa and Rudnicki, 2010). The final model variables were determined by taking the intersection of the variables selected by both the LASSO and Boruta methods, ensuring that only the most significant and robust predictors, which comprehensively reflect patient outcomes, were included.

#### Collinearity assessment

To evaluate the presence of multicollinearity among the selected variables, the Variance Inflation Factor (VIF) was computed. Variables with a VIF value exceeding 5 were excluded from the model to mitigate the adverse effects of multicollinearity on the regression analysis (Vatcheva et al., 2016).

#### Model construction

A nomogram was developed using the selected variables to predict 28-day all-cause mortality for patients with BSI. The nomogram incorporated a comprehensive set of demographic characteristics and clinical variables, including age, albumin levels, BUN, use of CRRT, GCS, lactate levels, mechanical ventilation status, presence of a malignant tumor, PTT, platelet count, RDW, and vasopressor use. Each variable was assigned a point value based on its relative contribution to the prediction of mortality risk, allowing for a quantitative assessment of individual patient risk.

#### Model evaluation

The discriminative ability of the nomogram and the SOFA score was evaluated by assessing the area under the receiver operating characteristic curve (AUROC). The performance improvement of the nomogram compared to the SOFA score, APSIII score, and SAPS II was assessed using the Integrated Discrimination Improvement (IDI) and the Net Reclassification Improvement (NRI). Calibration curves and the Hosmer-Lemeshow test were utilized to evaluate the calibration of the nomogram. The net clinical benefit was determined through the decision curve analysis (DCA) curve.

#### Model interpretation

To quantify the importance of each variable in the model, the SHAP (SHapley Additive exPlanations) method was employed. SHAP values provide a measure of the contribution of each feature to the prediction, allowing for the interpretation of the model’s output in terms of the impact of individual variables (Garriga et al., 2022).

#### Data analysis

The data distribution was analyzed using the Shapiro–Wilk test. Continuous data were represented as mean ± standard deviation or median (interquartile range, IQR), while categorical variables were presented as frequencies and ratios (%). Non-parametric tests (Mann–Whitney U test or Kruskal-Wallis test) were employed for non-normally distributed or heteroscedastic data. Pearson’s chi-square test was used to compare categorical data. All statistical analyses were carried out using R software, utilizing various packages including tableone, mice, rms, pROC, dca, and rdma.

## Results

### Baseline characteristics

We included 3,615 patients with positive blood cultures, 2,532 in the training cohort and 1,083 in the validation cohort. In the training cohort, 71.8% of patients survived, while 28.2% died. Non-survivors were older (median age 69.0 years [IQR, 59.0-79.0] *vs* 64.0 years [IQR, 52.0-74.0]; P<0.001) and had higher prevalence of myocardial infarction (11.5% *vs* 7.8%; P=.004), congestive heart failure (35.2% *vs* 28.7%; P=0.001), chronic obstructive pulmonary disease (10.3% *vs* 7.2%; P=0.009), malignant tumor (20.3% *vs* 12.1%; P<0.001), chronic kidney disease (29.7% *vs* 19.2%; P<0.001), and cirrhosis (16.2% *vs* 10.1%; P<0.001). Initial vital signs and laboratory findings showed that non-survivors had lower systolic blood pressure (114.0 mm Hg [IQR, 98.0-132.0] *vs* 117.0 mm Hg [IQR, 101.0-138.0]; P=0.001) and temperature (36.78°C [IQR, 36.44-37.17] *vs* 36.89°C [IQR, 36.56-37.33]; P<0.001), and higher levels of lactate (2.8 mmol/L [IQR, 1.7-4.9] *vs* 1.8 mmol/L [IQR, 1.2-2.8]; P<0.001), creatinine (1.8 mg/dL [IQR, 1.1-3.2] *vs* 1.2 mg/dL [IQR, 0.8-2.1]; P<0.001), and BUN (39 mg/dL [IQR, 24-63] *vs* 25 mg/dL [IQR, 16-41]; P<0.001). Similar patterns were observed in the validation cohort (**Table 1**).

**Table 1:** Baseline characteristics and comparison of training and validation cohorts.

Characteristics	Training cohort (N=2532)			Validation cohort (N=1083)			P
	Survival (N=1817)	Non-survival (N=715)	P	Survival (N=795)	Non-survival (N=288)	P	
**Age** (years)	64.00 (52.00, 74.00)	69.00 (59.00, 79.00)	<0.001	64.00 (53.00, 73.00)	71.00 (61.00, 79.00)	<0.001	0.730
**BMI**	28.40 (24.04, 33.52)	27.40 (23.75, 33.33)	0.040	28.92 (24.53, 33.69)	27.39 (23.13, 33.07)	0.024	0.551
**Gender,** n (%)			0.965			0.591	0.818
Male	1091 (60.0%)	430 (60.1%)		477 (60.0%)	178 (61.8%)		
Female	726 (40.0%)	285 (39.9%)		318 (40.0%)	110 (38.2%)		
**Race,** n (%)			0.013			0.648	0.832
White	1173 (64.6%)	443 (62.0%)		495 (62.3%)	176 (61.1%)		
Black	223 (12.3%)	70 (9.8%)		104 (13.1%)	31 (10.8%)		
Asin	59 (3.2%)	18 (2.5%)		28 (3.5%)	9 (3.1%)		
Hispanic or Latino	75 (4.1%)	34 (4.8%)		35 (4.4%)	15 (5.2%)		
Others	287 (15.8%)	150 (21.0%)		133 (16.7%)	57 (19.8%)		
Comorbidities							
Myocardial infarct, n (%)			0.004			0.550	0.986
No	1675 (92.2%)	633 (88.5%)		727 (91.4%)	260 (90.3%)		
Yes	142 (7.8%)	82 (11.5%)		68 (8.6%)	28 (9.7%)		
Congestive heart failure, n (%)			0.001			0.089	0.181
No	1296 (71.3%)	463 (64.8%)		546 (68.7%)	182 (63.2%)		
Yes	521 (28.7%)	252 (35.2%)		249 (31.3%)	106 (36.8%)		
COPD, n (%)			0.009			0.949	0.500
No	1686 (92.8%)	641 (89.7%)		725 (91.2%)	263 (91.3%)		
Yes	131 (7.2%)	74 (10.3%)		70 (8.8%)	25 (8.7%)		
Diabetes Mellitus, n (%)			0.495			0.612	0.169
No	1190 (65.5%)	458 (64.1%)		502 (63.1%)	177 (61.5%)		
Yes	627 (34.5%)	257 (35.9%)		293 (36.9%)	111 (38.5%)		
Malignant tumor, n (%)			<0.001			0.031	0.702
No	1598 (87.9%)	570 (79.7%)		688 (86.5%)	234 (81.2%)		
Yes	219 (12.1%)	145 (20.3%)		107 (13.5%)	54 (18.8%)		
CKD, n (%)			<0.001			0.011	0.712
No	1468 (80.8%)	503 (70.3%)		630 (79.2%)	207 (71.9%)		
Yes	349 (19.2%)	212 (29.7%)		165 (20.8%)	81 (28.1%)		
Hypertension, n (%)			0.015			0.677	0.778
No	1163 (64.0%)	494 (69.1%)		527 (66.3%)	187 (64.9%)		
Yes	654 (36.0%)	221 (30.9%)		268 (33.7%)	101 (35.1%)		
Cirrhosis, n (%)			<0.001			<0.001	0.069
No	1633 (89.9%)	599 (83.8%)		702 (88.3%)	229 (79.5%)		
Yes	184 (10.1%)	116 (16.2%)		93 (11.7%)	59 (20.5%)		
Hyperlipidemia, n (%)			0.510			0.256	0.477
No	1257 (69.2%)	485 (67.8%)		564 (70.9%)	194 (67.4%)		
Yes	560 (30.8%)	230 (32.2%)		231 (29.1%)	94 (32.6%)		
Microorganism, n (%)			0.317			0.422	0.790
Gram-positive cocci	1151 (63.3%)	441 (61.7%)		501 (63.0%)	186 (64.6%)		
Gram-positive rods	85 (4.7%)	27 (3.8%)		32 (4.0%)	8 (2.8%)		
Gram-negative rods	427 (23.5%)	194 (27.1%)		198 (24.9%)	74 (25.7%)		
Gram-negative cocci	150 (8.3%)	52 (7.3%)		64 (8.1%)	19 (6.6%)		
Fungi	4 (0.2%)	1 (0.1%)		0 (0.0%)	1 (0.3%)		
First day vital signs (IQR)							
Heart rate (min-1)	95.00 (80.00, 111.00)	96.00 (81.00, 111.00)	0.393	95.00 (80.00, 111.00)	95.00 (80.00, 114.00)	0.616	0.780
Sbp (mmHg)	117.00 (101.00, 138.00)	114.00 (98.00, 132.00)	0.001	117.00 (101.00, 137.00)	112.00 (97.25, 134.75)	0.023	0.882
Dbp (mmHg)	67.00 (56.00, 79.00)	65.00 (53.00, 78.00)	0.034	65.00 (54.00, 79.00)	62.00 (53.00, 79.00)	0.099	0.169
Mbp (mmHg)	79.00 (68.00, 92.00)	77.00 (66.00, 91.00)	0.010	79.00 (68.00, 93.00)	76.50 (65.00, 90.75)	0.130	0.504
RR (min-1)	20.00 (16.00, 25.00)	21.00 (17.00, 25.00)	0.014	20.00 (16.00, 24.00)	21.00 (17.00, 26.00)	0.011	0.757
Temperature (°C)	36.89 (36.56, 37.33)	36.78 (36.44, 37.17)	<0.001	36.89 (36.56, 37.33)	36.72 (36.39, 37.21)	<0.001	0.757
SpO2 (%)	98.00 (95.00, 100.00)	97.00 (94.00, 99.00)	<0.001	98.00 (95.00, 100.00)	97.00 (95.00, 100.00)	0.494	0.159
First day laboratory tests (IQR)							
Hemoglobin (g/dL)	10.10 (8.45, 11.80)	9.60 (8.10, 11.10)	<0.001	10.10 (8.60, 11.80)	9.65 (8.30, 11.40)	0.016	0.474
Platelets (K/uL)	182.00 (117.00, 254.00)	160.00 (88.00, 236.00)	<0.001	183.00 (121.00, 259.00)	157.00 (92.25, 233.75)	<0.001	0.553
WBC (K/uL)	11.60 (7.70, 16.50)	11.90 (7.50, 18.00)	0.351	11.90 (8.00, 17.80)	12.20 (7.95, 17.48)	0.996	0.160
RBC (m/uL)	3.39 (2.87, 4.00)	3.19 (2.67, 3.82)	<0.001	3.43 (2.90, 3.98)	3.23 (2.73, 3.82)	0.001	0.360
RDW (%)	15.00 (13.70, 16.70)	16.00 (14.60, 18.40)	<0.001	15.00 (13.80, 16.90)	15.95 (14.60, 17.88)	<0.001	0.487
Albumin (g/dL)	2.80 (2.40, 3.30)	2.70 (2.20, 3.10)	<0.001	2.80 (2.40, 3.20)	2.60 (2.20, 3.10)	<0.001	0.293
Creatinine (mg/dL)	1.10 (0.80, 1.80)	1.40 (0.90, 2.50)	<0.001	1.10 (0.80, 2.00)	1.60 (1.00, 2.80)	<0.001	0.147
BUN (mg/dL)	22.00 (14.00, 37.00)	31.00 (19.00, 54.00)	<0.001	22.00 (14.00, 40.00)	35.50 (21.00, 54.00)	<0.001	0.131
Chloride (mmol/L)	104.00 (99.00, 108.00)	102.00 (98.00, 107.00)	<0.001	103.00 (99.00, 108.00)	102.00 (98.00, 108.00)	0.348	0.707
Sodium (mmol/L)	138.00 (135.00, 141.00)	137.00 (133.00, 141.00)	0.022	138.00 (134.00, 141.00)	138.00 (134.00, 141.00)	0.797	0.379
Potassium (mmol/L)	4.10 (3.70, 4.60)	4.20 (3.70, 4.80)	<0.001	4.10 (3.70, 4.60)	4.20 (3.70, 4.90)	0.021	0.420
Bilirubin total (mg/dL)	0.70 (0.40, 1.50)	0.90 (0.50, 2.40)	<0.001	0.70 (0.40, 1.60)	0.95 (0.50, 2.27)	0.004	0.082
Glucose (mg/dL)	132.00 (106.00, 175.00)	131.00 (106.00, 185.00)	0.514	132.00 (107.00, 182.00)	139.00 (109.00, 198.75)	0.152	0.984
INR	1.30 (1.20, 1.70)	1.50 (1.20, 2.10)	<0.001	1.30 (1.20, 1.60)	1.60 (1.30, 2.00)	<0.001	0.830
PT (S)	14.80 (13.10, 18.10)	16.10 (13.50, 22.30)	<0.001	14.80 (13.00, 17.60)	17.10 (13.90, 22.40)	<0.001	0.952
PTT (S)	31.20 (27.30, 38.80)	35.70 (29.40, 49.10)	<0.001	31.20 (27.90, 38.80)	35.60 (28.90, 46.65)	<0.001	0.880
Lactate (mmol/L)	1.70 (1.10, 2.70)	2.20 (1.50, 3.80)	<0.001	1.80 (1.20, 2.70)	2.40 (1.50, 3.87)	<0.001	0.056
pH	7.38 (7.32, 7.43)	7.36 (7.27, 7.43)	<0.001	7.38 (7.31, 7.44)	7.37 (7.28, 7.42)	0.040	0.836
PO2 (mmHg)	87.00 (52.00, 164.00)	79.00 (47.00, 131.00)	<0.001	83.00 (49.00, 146.00)	73.50 (47.25, 121.75)	0.019	0.686
PCO2 (mmHg)	40.00 (34.00, 46.00)	40.00 (33.00, 47.00)	0.790	39.00 (34.00, 46.00)	38.00 (32.00, 45.00)	0.037	0.102
ALT (IU/L)	30.00 (17.00, 65.00)	32.00 (17.00, 81.00)	0.165	29.00 (16.00, 62.00)	32.00 (16.25, 68.50)	0.309	0.307
AST (IU/L)	41.00 (24.00, 89.00)	51.00 (28.00, 136.00)	<0.001	40.00 (23.00, 82.00)	53.00 (28.00, 127.25)	<0.001	0.358
Sedative Medications							
Midazolam, n (%)			<0.001			0.142	0.921
No	1156 (63.6%)	399 (55.8%)		500 (62.9%)	167 (58.0%)		
Yes	661 (36.4%)	316 (44.2%)		295 (37.1%)	121 (42.0%)		
Propofol, n (%)			0.853			0.832	0.520
No	1699 (93.5%)	670 (93.7%)		740 (93.1%)	267 (92.7%)		
Yes	118 (6.5%)	45 (6.3%)		55 (6.9%)	21 (7.3%)		
Dexmedetomidine, n (%)			0.246			0.015	0.186
No	1334 (73.4%)	541 (75.7%)		556 (69.9%)	223 (77.4%)		
Yes	483 (26.6%)	174 (24.3%)		239 (30.1%)	65 (22.6%)		
Mechanical ventilationt, n (%)			<0.001			<0.001	0.959
No	906 (49.9%)	263 (36.8%)		391 (49.2%)	108 (37.5%)		
Yes	911 (50.1%)	452 (63.2%)		404 (50.8%)	180 (62.5%)		
CRRT, n (%)			<0.001			<0.001	0.890
No	1625 (89.4%)	519 (72.6%)		706 (88.8%)	213 (74.0%)		
Yes	192 (10.6%)	196 (27.4%)		89 (11.2%)	75 (26.0%)		
Vasopressor, n (%)			<0.001			<0.001	0.272
No	1023 (56.3%)	276 (38.6%)		431 (54.2%)	103 (35.8%)		
Yes	794 (43.7%)	439 (61.4%)		364 (45.8%)	185 (64.2%)		
Severe Score (IQR)							
Acute Physiology Score III	50.00 (38.00, 64.00)	67.00 (51.00, 84.00)	<0.001	51.00 (39.00, 65.00)	68.00 (54.00, 84.00)	<0.001	0.180
Simplified Acute Physiology Score II	38.00 (29.00, 47.00)	49.00 (38.00, 60.00)	<0.001	39.00 (30.00, 48.00)	49.50 (40.00, 58.00)	<0.001	0.076
SOFA score	5.00 (3.00, 8.00)	8.00 (5.00, 11.00)	<0.001	6.00 (3.00, 9.00)	8.00 (6.00, 12.00)	<0.001	0.020
Charlson Comorbidity Index	5.00 (3.00, 7.00)	7.00 (5.00, 9.00)	<0.001	5.00 (3.00, 8.00)	7.00 (5.00, 9.00)	<0.001	0.040
GCS	15.00 (13.00, 15.00)	14.00 (12.00, 15.00)	<0.001	15.00 (14.00, 15.00)	14.00 (12.00, 15.00)	<0.001	0.351
Outcome							
Length of hospital (days)	21.01 (11.86, 36.77)	13.91 (7.49, 23.36)	<0.001	23.90 (12.56, 40.55)	13.32 (6.49, 22.19)	<0.001	0.063
Length of ICU (days)	4.50 (2.12, 11.96)	5.41 (2.61, 11.25)	0.108	4.88 (2.28, 12.72)	5.03 (2.59, 10.61)	0.827	0.546
Hospital Mortality, n (%)			<0.001			<0.001	0.745
No	1730 (95.2%)	106 (14.8%)		746 (93.8%)	45 (15.6%)		
Yes	87 (4.8%)	609 (85.2%)		49 (6.2%)	243 (84.4%)		
ICU Mortality, n (%)			<0.001			<0.001	0.602
No	1779 (97.9%)	269 (37.6%)		773 (97.2%)	111 (38.5%)		
Yes	38 (2.1%)	446 (62.4%)		22 (2.8%)	177 (61.5%)		

Data are presented as median (interquartile range) for continuous variables and n (%) for categorical variables. P values in the last column compare training and validation cohorts, with P > 0.05 indicating no significant differences between groups.

BMI, Body Mass Index; CKD, Chronic Kidney Disease; COPD, Chronic Obstructive Pulmonary Disease; WBC, White Blood Cell; RBC, Red Blood Cell; RDW, Red Cell Distribution Width; INR, International Normalized Ratio; PT, Prothrombin Time; PTT, Partial Thromboplastin Time; SOFA, Sequential Organ Failure Assessment; GCS, Glasgow Coma Scale; ICU, Intensive Care Unit; CRRT, Continuous Renal Replacement Therapy; SpO2, Peripheral Capillary Oxygen Saturation; Sbp, Systolic Blood Pressure; Dbp, Diastolic Blood Pressure; Mbp, Mean Blood Pressure.

### Model development and variable selection

**Figure 2:**
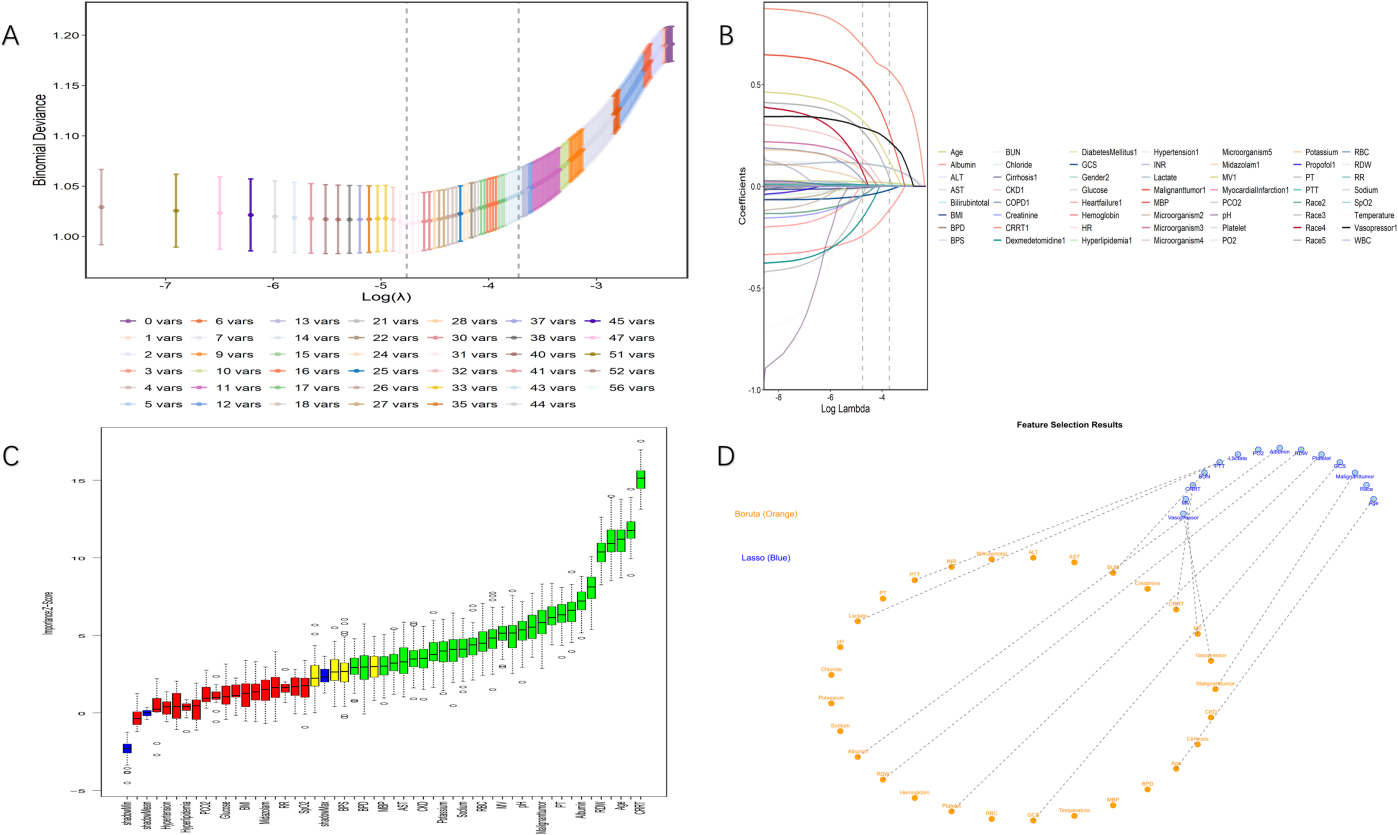
Process and results of variable selection. **(A)** Selection of tuning parameter (lambda) in LASSO regression using minimum criteria (left dotted line) and 1-SE criteria (right dotted line). **(B)** Coefficient distribution created from the log(lambda) sequence. In this study, predictor variables were selected based on the 1-SE criterion (right dotted line), resulting in 14 nonzero coefficients. **(C)** Importance scores of predictor variables calculated by the Boruta algorithm. The vertical axis represents the importance score in Z-score form, while the horizontal axis lists all predictor variables. **(D)** Feature selection results showing key variables identified by both Boruta algorithm (orange) and LASSO regression (blue). The final selected variables represent the intersection of both methods, providing high-confidence predictors for the model.LASSO indicates least absolute shrinkage and selection operator; SE, standard error.

**Figure 3:**
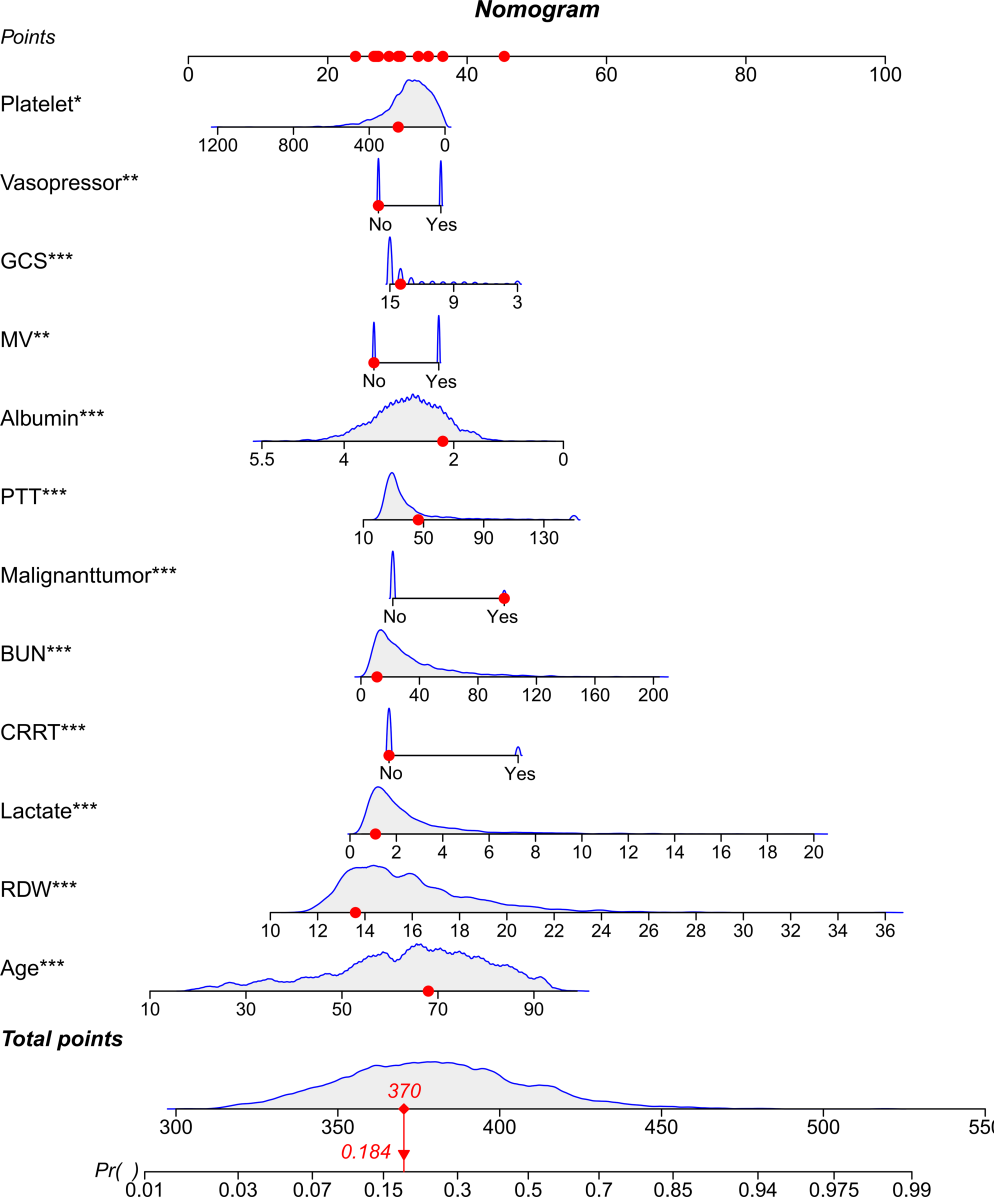
Nomogram for predicting the outcome. Nomogram for estimating the probability of the outcome based on selected clinical variables. Each variable contributes points that sum to a total score, which corresponds to the predicted probability on the bottom scale. *p < 0.05; **p < 0.01; ***p < 0.001.

Through a two-step variable selection process combining LASSO regression and Boruta algorithm, we identified 12 predictive variables from the initial set of clinical parameters. LASSO regression initially selected 14 variables (**Figures 2A, B**), while Boruta algorithm confirmed 30 important features (**Figure 2C**). The intersection of these methods yielded the final 12 variables: age, albumin, BUN, CRRT, GCS, lactate, mechanical ventilation, malignant tumor, PTT, platelet count, RDW, and vasopressor use (**Figure 2D**). Multicollinearity assessment demonstrated variance inflation factor values below 2 (range, 1.02-1.29) for all selected variables, indicating minimal collinearity. Based on these variables, we constructed a nomogram to predict 28-day all-cause mortality for patients with BSI (**Figure 3**). The nomogram incorporated both demographic characteristics and clinical variables, with point values assigned to each predictor based on their relative contribution to mortality risk.

### Predictive model performance

**Figure 4:**
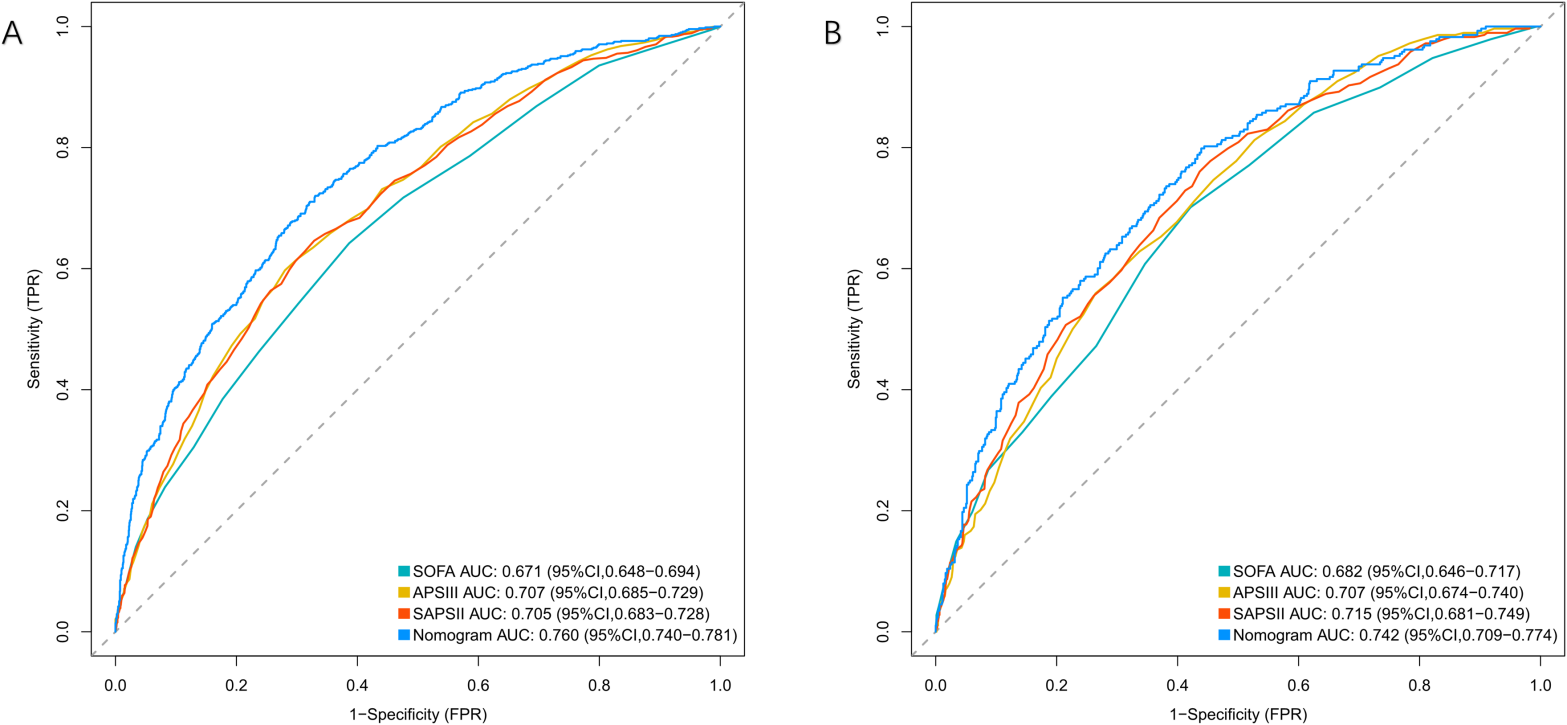
ROC curves for predicting 28-day mortality in patients with bloodstream infections. **(A)** Training Cohort and **(B)** Validation Cohort compare the performance of the Nomogram, SOFA, APSIII, and SAPSII scores. The Nomogram demonstrates superior predictive ability in both cohorts.

The nomogram demonstrated superior discrimination (AUROC, 0.760 [95% CI, 0.740-0.781]) compared with SOFA (0.671 [0.648-0.694]), APSIII (0.705 [0.683-0.728]), and SAPS II (0.707 [0.685-0.729]) (all P<0.001) in the training cohort. In the validation cohort, the nomogram (AUROC, 0.742 [95% CI, 0.709-0.775]) maintained significantly better discrimination than SOFA (0.681 [0.645-0.717], P=0.001) and SAPS II (0.701 [0.665-0.737], P=0.038), although the difference with APSIII (0.715 [0.680-0.750], P=0.129) did not reach statistical significance (**Figure 4**, **Table 2**).

**Table 2:** Comparison of the performance of four models in predicting 28-day all-cause mortality in patients with positive blood cultures.

Predict Model		AUROC	*P* value	NRI (Categorical)	*P* value	NRI (Continuous)	*P* value	IDI	*P* value
Training set	Nomogram	0.760							
	SOFA	0.671	<0.001	0.1422 [0.097-0.1873]	<0.001	0.5859 [0.5023-0.6683]	<0.001	0.1017 [0.0869-0.1164]	<0.001
	APSIII	0.705	<0.001	0.0943 [0.054-0.1346]	<0.001	0.442 [0.3574-0.5266]	<0.001	0.0735 [0.0581-0.089]	<0.001
	SAPSII	0.707	<0.001	0.0758 [0.0375-0.114]	<0.001	0.4175 [0.3325-0.5025]	<0.001	0.07 [0.055-0.085]	<0.001
Validation set	Nomogram	0.742							
	SOFA	0.681	0.001	0.0809 [0.0041-0.1577]	0.039	0.4964 [0.3653-0.6275]	<0.001	0.084 [0.0591-0.1089]	<0.001
	APSIII	0.715	0.129	0.0772 [0.0139-0.1406]	0.017	0.3251 [0.192-0.4582]	<0.001	0.0592 [0.0343-0.0841]	<0.001
	SAPSII	0.701	0.038	0.0697 [0.0082-0.1312]	0.026	0.3721 [0.2391-0.5051]	<0.001	0.0633 [0.0388-0.0878]	<0.001

The P-value was calculated by comparing the results of the nomogram with SOFA, APSIII, and SAPSII.

SOFA, Sequential Organ Failure Assessment. APSIII, Acute Physiology and Chronic Health Evaluation III. SAPSII, Simplified Acute Physiology Score II. AUROC, Area Under the ROC Curve. NRI, Net Reclassification Improvement. IDI, Integrated Discrimination Improvement.

### Calibration and model reclassification

**Figure 5:**
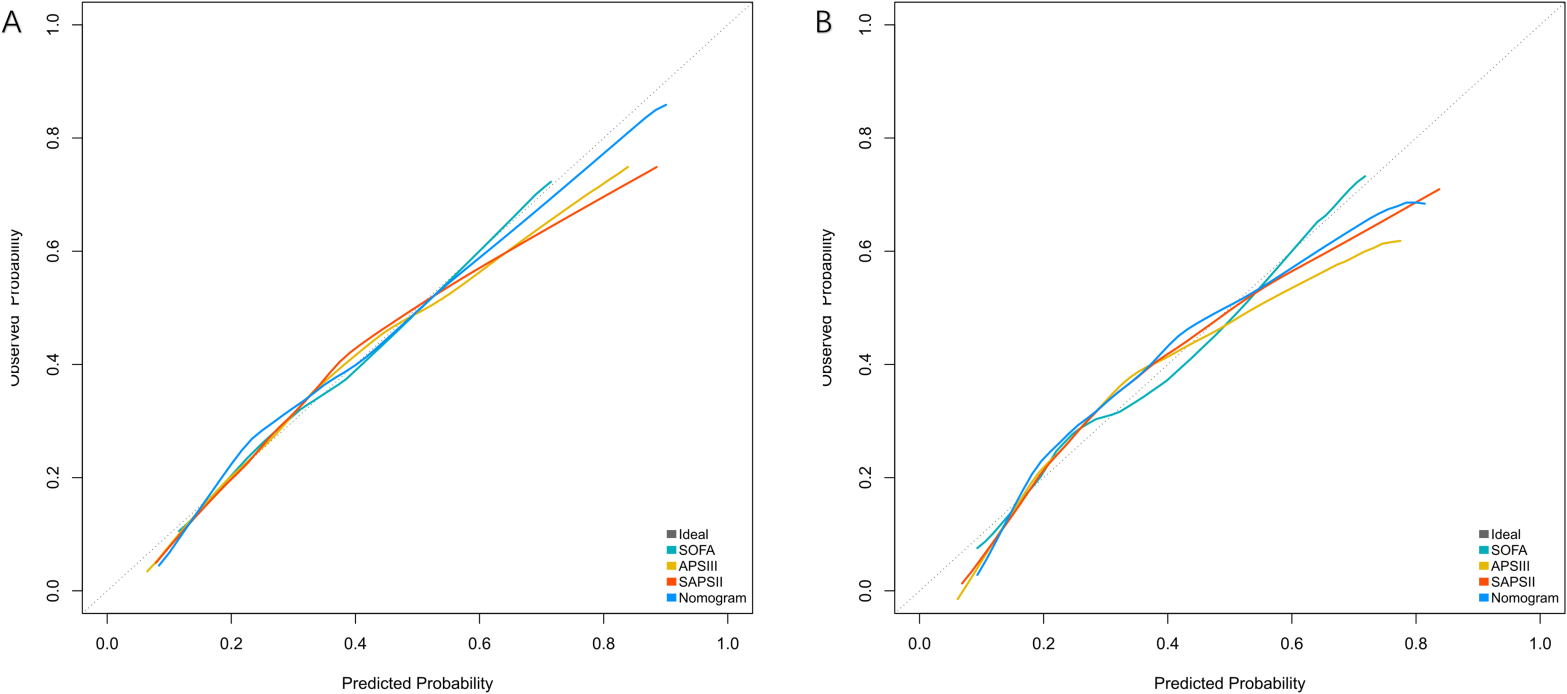
Calibration curves for predicting 28-day mortality in patients with bloodstream infections. **(A)** Training Cohort and **(B)** Validation Cohort compare the predicted probabilities of the Nomogram, SOFA, APSIII, and SAPSII scores against the observed probabilities. The dashed line represents the ideal calibration (perfect agreement between predicted and observed probabilities). The Nomogram shows the closest alignment to the ideal line in both cohorts, indicating better calibration performance.

Calibration was assessed using the Hosmer-Lemeshow test and calibration curves. The Hosmer-Lemeshow test showed good calibration in both the training (χ²=12.39, df=6, P=0.054) and validation cohorts (χ²=11.576, df=6, P=0.072), indicating no significant deviation between predicted and observed outcomes. The calibration curves demonstrated good agreement between predicted and actual probabilities across the entire range of predicted risk (**Figure 5**).

**Figure 6:**
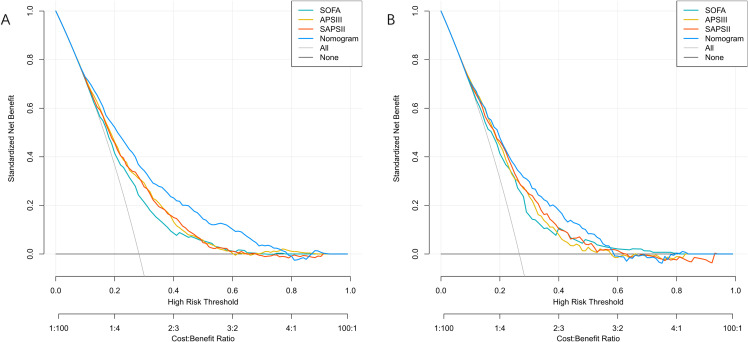
Decision curve analysis for predicting 28-day mortality in patients with bloodstream infections. **(A)** Training Cohort and **(B)** Validation Cohort compare the net benefit of the Nomogram, SOFA, APSIII, and SAPSII scores. The Nomogram shows higher clinical utility across a wider range of threshold probabilities in both cohorts.

**Figure 7:**
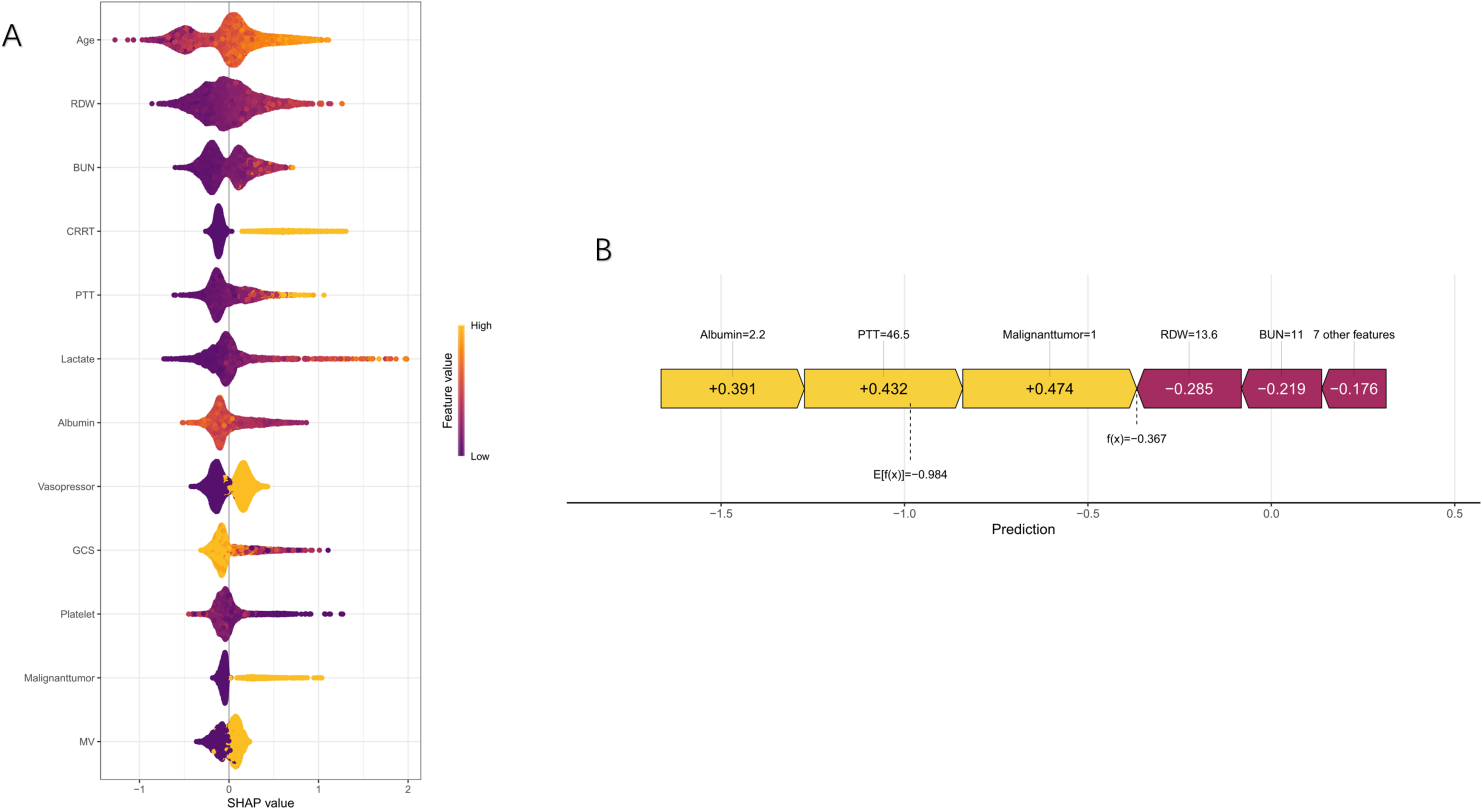
SHAP analysis for predicting 28-day mortality in bloodstream infection patients. The beeswarm plot **(A)** shows the distribution of SHAP values for each feature, with color intensity indicating feature values. The force plot **(B)** illustrates the contribution of individual features to a specific prediction, showing how each feature affects the model’s output.

The nomogram showed significant improvements in risk reclassification compared with conventional scores. In the training cohort, categorical NRI values were 0.1422 (95% CI, 0.097-0.1873) versus SOFA, 0.0943 (0.054-0.1346) versus APSIII, and 0.0758 (0.0375-0.114) versus SAPS II (all P<0.001). Continuous NRI values showed similar improvements: 0.5859 (0.5023-0.6683) versus SOFA, 0.442 (0.3574-0.5266) versus APSIII, and 0.4175 (0.3325-0.5025) versus SAPS II (all P<0.001) (**Table 2**). Decision curve analysis demonstrated consistent net benefit across a wide range of threshold probabilities (0.08-0.92 in training; 0.10-0.84 in validation cohorts) (**Figure 6**). SHAP analysis identified mechanical ventilation, malignancy, platelet count, and GCS as the strongest predictors of mortality (**Figure 7**).

## Discussion

In this cohort study of 3,615 patients with BSI, we developed and validated a predictive model for 28-day all-cause mortality that demonstrated superior performance compared with conventional severity scores, such as SOFA, APSIII, and SAPS II. By integrating 12 key clinical and laboratory variables spanning multiple pathophysiological domains, the model highlights the importance of a multidimensional approach to risk stratification in sepsis. These findings underscore the critical role of combining metabolic, neurological, and immunological indicators with therapeutic interventions to enhance prognostic accuracy.

The superior performance of our model can be attributed to several factors. First, the inclusion of both laboratory and clinical variables provided a more comprehensive assessment of disease severity than traditional scoring systems. SHAP analysis revealed that mechanical ventilation, malignancy, and platelet count were among the strongest predictors, emphasizing the importance of combining intervention requirements, comorbidity burden, and physiological derangements to better capture mortality risk. Second, the model was robustly validated, demonstrating strong discriminative ability in both training and validation cohorts, supporting its potential generalizability across similar populations. The clinical utility of the model is further underscored by its superior performance compared with conventional severity scores and its ability to improve risk stratification across the spectrum of disease severity. Metrics such as NRI and IDI demonstrated significant enhancements in risk prediction, while DCA confirmed consistent net benefit across a wide range of threshold probabilities. These results suggest the model could serve as a valuable tool for early risk stratification, guiding clinical decision-making, and optimizing resource allocation in patients with BSI.

The identified predictors align with current understanding of sepsis pathophysiology. Neurological dysfunction, represented by the GCS, emerged as a key determinant of mortality. Lower GCS scores, indicative of sepsis-associated encephalopathy (SAE) (Jin et al., 2024), were strongly associated with poor outcomes (Bourhy et al., 2022; Fang et al., 2022; Zou et al., 2022), consistent with prior studies highlighting the prognostic significance of neurological status in sepsis. Elevated lactate levels, a marker of tissue hypoperfusion and metabolic dysfunction, were similarly predictive of mortality. Lactate and lactate clearance in acute cardiac care patients, Occurrence and adverse effect on outcome of hyperlactatemia in the critically ill (Khosravani et al., 2009; Attana et al., 2012; Wright et al., 2022), reinforcing their established role as a key indicator of disease severity. Other laboratory predictors, including hypoalbuminemia, elevated BUN, thrombocytopenia, and prolonged PTT, reflect the systemic derangements characteristic of sepsis. These findings align with known mechanisms where hypoalbuminemia signals systemic inflammation and malnutrition (Furukawa et al., 2019; Mahmud et al., 2021), elevated BUN reflects renal dysfunction (Hu et al., 2021; Harazim et al., 2023; Li et al., 2024), and thrombocytopenia and coagulopathy are markers of disseminated intravascular coagulation (DIC) and severe systemic inflammation (Valladolid et al., 2020; Harmon et al., 2021; Jahn et al., 2022). Notably, the prolonged PTT underscores coagulopathic changes, acting as an indicator of the severity of coagulopathy in sepsis and its correlation with poorer clinical outcomes (Guo et al., 2022). Furthermore, the inclusion of RDW enriches this discussion. Elevated RDW levels suggest increased inflammation and oxidative stress within the body, positioning RDW as a significant prognostic marker that indicates a heightened risk of mortality in patients with sepsis (Crook et al., 2022; Wu et al., 2022). Collectively, these variables offer a more nuanced understanding of the complex pathophysiology of sepsis and its implications for mortality risk, emphasizing the necessity of monitoring these parameters in clinical practice.

The inclusion of therapeutic interventions as predictors—mechanical ventilation, continuous renal replacement therapy, and vasopressor use—merits particular attention. While these variables may partly reflect disease severity, their independent contribution to the model suggests they capture unique aspects of the clinical trajectory not fully represented by physiological parameters alone (Baghdadi et al., 2020; Evans et al., 2021; Bhavani et al., 2022). The SHAP analysis highlighted the substantial impact of these interventions on the model’s predictions, suggesting that treatment-related variables may serve as critical markers of disease progression and prognosis. However, careful interpretation is needed to distinguish between markers of severity and potentially modifiable risk factors.

Comorbidities also played a significant role in mortality prediction. Malignancy, in particular, emerged as a strong predictor, likely reflecting the immunosuppressive effects of both the disease and its treatments (Danahy et al., 2019; Hensley et al., 2019; Cooper et al., 2020). The SHAP analysis underscored the substantial contribution of malignancy to the model’s predictive power, highlighting the importance of accounting for comorbid conditions in risk stratification for BSI patients.

In our study, we compared our nomogram model with three traditional severity scores—SOFA, APSIII, and SAPS II—commonly used to assess critically ill patients but limited in predicting mortality in BSI (Vincent and Moreno, 2010).The SOFA score, while a cornerstone for evaluating organ dysfunction, relies on a narrow set of physiological parameters and excludes key factors like comorbidities, treatment interventions, and laboratory markers, reducing its predictive accuracy for BSI-related mortality (Gershengorn et al., 2021). Similarly, APSIII and SAPS II, though incorporating more variables, fail to address the unique pathophysiology of BSI, omitting critical predictors such as mechanical ventilation, malignancy, platelet count, and lactate levels (Le Gall et al., 1993; Nassar et al., 2014). In contrast, our nomogram model adopts a multidimensional approach, integrating demographic, clinical, laboratory, and treatment variables to provide a more comprehensive assessment of disease severity. This holistic design captures the complex interplay of factors influencing mortality, significantly enhancing predictive accuracy and outperforming traditional scores.

This study has several strengths. The use of a large, well-characterized dataset and robust statistical methods for variable selection and model validation enhances the reliability and generalizability of the findings. By integrating diverse clinical and laboratory variables, the model achieves improved discriminatory power and clinical relevance compared with existing severity scores. Several limitations must be acknowledged. The retrospective design and reliance on data from a single healthcare system may limit the generalizability of our findings. Additionally, our model does not account for dynamic changes in variables over time, which could enhance risk prediction. To address these limitations, future research should focus on validating the model prospectively across diverse populations and incorporating longitudinal data to improve predictive accuracy. Furthermore, studies should explore the causal relationships between key predictors and outcomes, identifying modifiable factors for targeted interventions. Incorporating serial measurements of critical variables, such as lactate and platelets, could enhance the model’s ability to capture the evolving clinical trajectory of sepsis, paving the way for more personalized approaches to risk assessment and management for patients with BSI.

## Conclusion

This study developed and validated a predictive model for 28-day all-cause mortality in patients with BSI, demonstrating superior performance compared to traditional severity scores. By integrating clinical, laboratory, and treatment-related variables, the model provides a more comprehensive approach to risk stratification. These findings highlight its potential for improving early identification of high-risk patients and guiding clinical decision-making, though further prospective validation is needed to confirm its generalizability.

## Data Availability

The study utilized data sourced from the Medical Information Mart for Intensive Care IV (MIMIC-IV) database. Access to this dataset is restricted to credentialed users who have completed the necessary training (e.g., CITI Data or Specimens Only Research) and signed the data use agreement. Researchers interested in accessing the data can submit requests through PhysioNet at https://physionet.org/.
