# Supplementary figures and images for: Development and validation of a multidimensional predictive model for 28-day mortality in ICU patients with bloodstream infections: a cohort study
Source: Front Cell Infect Microbiol. 2025 Jul 7;15:1569748. doi: 10.3389/fcimb.2025.1569748 (PMC12277296; doi:10.3389/fcimb.2025.1569748)

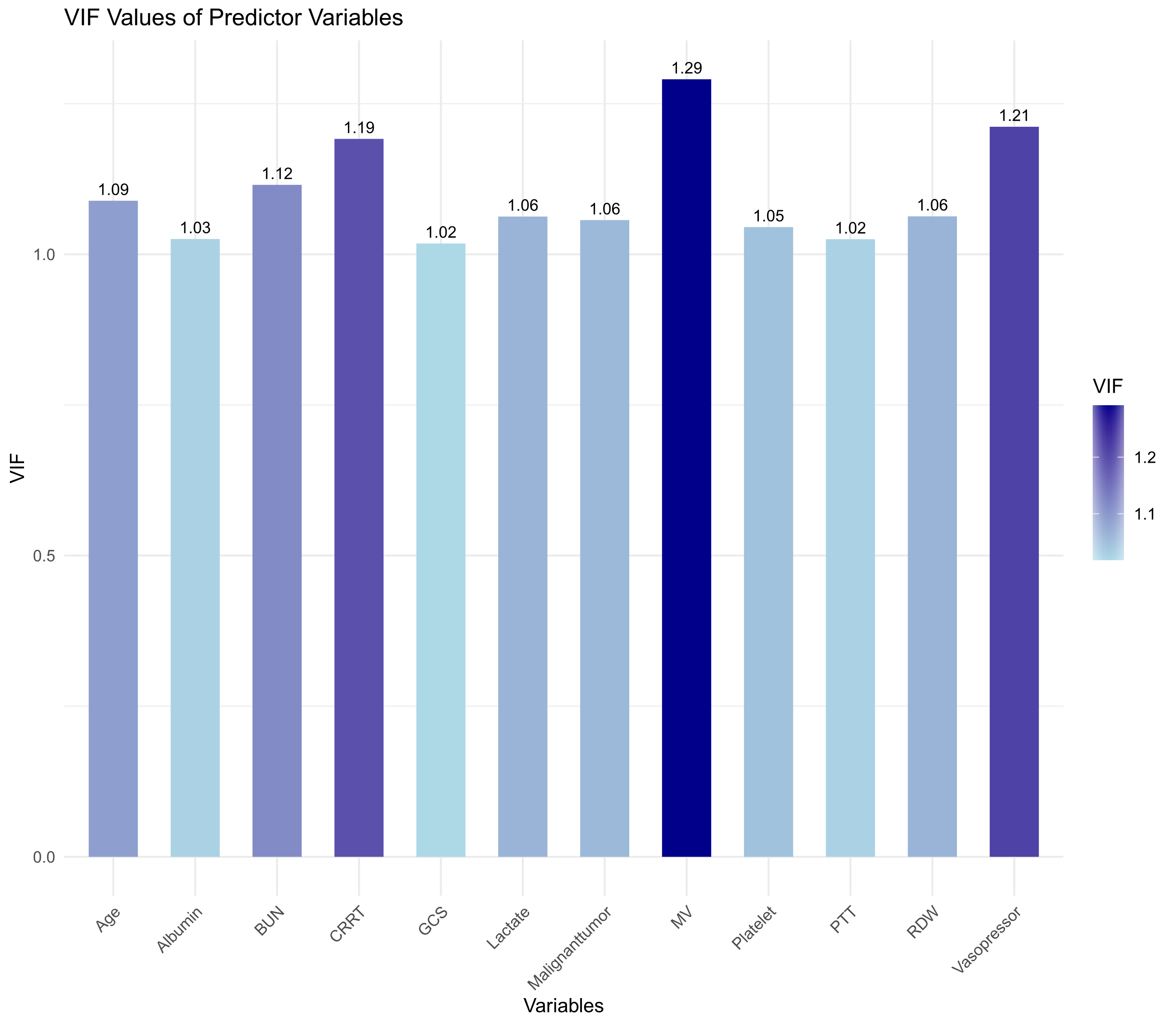

Supplement: Supplementary file 2 [file Image1.tif]

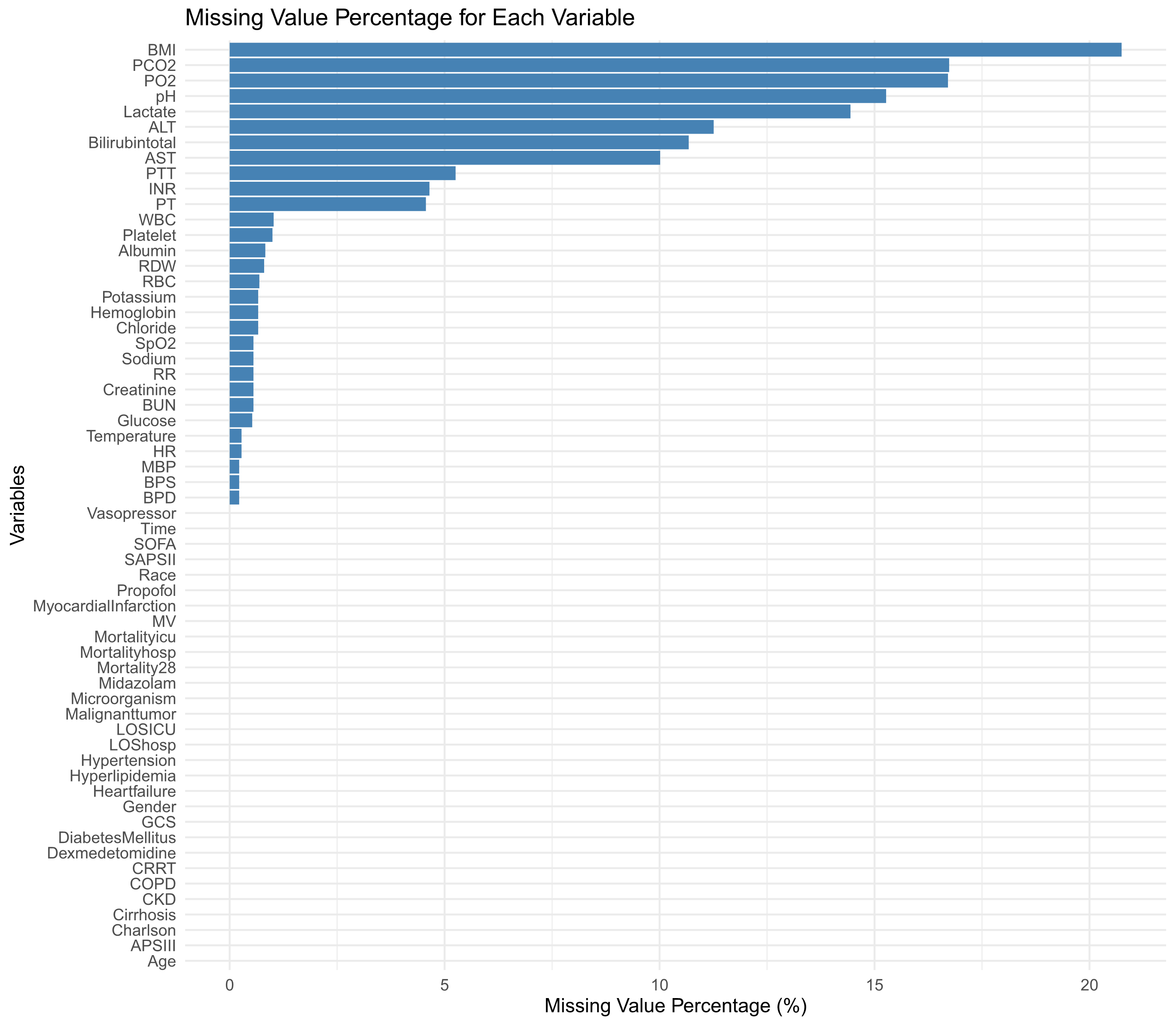

Supplement: Supplementary file 3 [file Image2.tif]
